# Selections

**Published:** 1881-09

**Authors:** 


### SELECTIONS.

### ON THE TREATMENT OF YELLOW FEVER.

BY RICHARD H. DAY, M. D.

Now, with this state of feeling pervading all classes, what are likely to be necessary consequences, when scores are prostrated by this rapid and malignant disease, unless this fear and trepidation are counteracted by some appropriate and adequate means? We all too well know the result. Right here, then, is the beginning point in the correct treatment of yellow fever. Disarm your patient, at your very first visit, of his fears and apprehensions of a fatal result. Inspire confidence and infuse into him moral courage and mental vigor.

This, as his physician, you should be able to do. When your patient watches your every move and expression; looks you in the eyes, and with despair depicted in his countenance, trembing asks you, "Doctor, do you think I can get well?" Tell him with emphasis, yes, if you will be a man, and dismiss your unnecessary and foolish fears, that the disease you are now suffering with is not *necessarily* as dangerous as pneumonia, nor as difficult to treat as pernicious, remittent and intermittent fevers. Tell him this, believing it, and say it in such a manner as will convince him that you do believe it. You will thus, kindle up hope; create as it were a renewed vitality of mental action, and this, acting upon the general nervous system, produces a corresponding better condition of all the functions of the animal mechanism, thus rendering his situation more favorable for judicious medication. Sometimes you will find a patient whose despair has plunged him into the slough of indifference and listlessness. He turns his face from you when you approach him, as though you were an intruder, and scarcely takes interest enough in himself to answer your questions. These are more difficult to manage than the former. But you must endeavor to incite them to hope; work to dispel that indifference, and to wake up their beclouded minds to a cheerful hope of life. For this, the medical attendant must be well imbued with a sense of his responsibility and the high mission of his calling, and requires a well trained and disciplined mind and high moral courage. That strong impressions made upon the mind in sickness, as in health, do exercise a powerful and controlling influence over the functions of the animal economy, I presume no man, posted in the literature of his profession, and not defective in observation, will deny ; and it this potent and acknowledged principle in the science of life, which it is the duty of every physician to understand and apply when occasion requires it. Having, then, invoked this principle in these cases, where needed, to the best of our ability, we turn our attention to the other condiitons. And the first thing that we have to comprehend, and to bear constantly in mind, when we are called to treat this disease, whether it be mild or grave in its manifestations, is the undisputed fact, that we have to contend with a train of morbid actions, that are rapid in their progress and vicious in their tendencies. And hence, whatever measures are adopted of a curative character, must be promptly resorted to ; used at the very incipiency of the attack, at least within the first twelve or twenty-four hours, before those chemical and moleeular changes take place in the organic structures and fluids of the system, that render a cure impossible.

Bearing this general and fundamental fact in mind, we must treat each case according to the special indications present. If the skin is hot and dry, or dry without being hot, as is sometimes the case, the patient, being in bed, should have a warm or hot mustard foot-bath, given under blankets to retain the vapor supplemented by potions of any warm diaphoretic beverage, in order to determine the circulation to the skin, and to induce a moderate diaphoresis, thus to relieve in some measure the interal congested organs. But this sweating process must not be carried to excess, either in quantity or duration of time, which is almost universally done, to the great discomfort and disadvantage of the patient. When the function of the skin is once established, it can be easily maintained at the proper standard, and still keep the patient comfortable, by giving small and repeated potations of cold or iced water—allowing free ventilation of the room, and a light blanket or two, according to temperature, to prevent any sudden cooling impression upon the cutaneous surface.

Should the seizure occur when the stomach is loaded with food, it should at once be emptied, either with warm water, mustard and salt, or ipecacuanha ; and the stomach then quieted as quickly as possible, should it be left irritable. For this purpose a mustard cataplasm applied over the epigastrium, and a little mint tea, mint julep, or small doses of morphine with bi-carb. soda given internally, will suffice. Frequently merely sponging the face and temples with cologne, bay rum or eau sedative, will produce a satisfactory revulsive effect.

Sometimes, at the onset, the bowels are loaded and constipated. Here it will be expedient and necessary to unload them ; but it is by no means a matter of indifference by what means it is done. I carefully discard and condemn the use of *castor oil*, which is the standard and almost universal cathartic in every case in the hands of practitioners and trained nurses. And I discard it, as I do every other irritating cathartic, from its well-known irritating properties upon the mucous coat of the stomach and bowels, and its further aptitude to produce a persistent nausea. With this property of the drug so well established and clearly manifested, in almost every case in which it is administered, it is a matter of extreme surprise that physicians should so pertinaciously cling to its use, and more especially since in this disease irritability and disturbance of the stomach are so prone to occur and so much to be dreaded. Instead of castor oil, enemas of warm water with camphorated oil should be given, which will always empty the bowels without irritating or disturbing the nervous filaments or mucous coat of the stomach and bowels. Should a more active cathartic be required, as is sometimes, but rarely the case, to work off the vitiated secretions, and to unload the mucous follicles and intestinal capillaries, an infusion of ʒ ii fol. sennæ with an ounce or ounce and a half of sulphate magnesia, combined with some agreeable aromatic to cover the taste, and given in divided doses, will answer an admirable purpose.

Sometimes the beginning of the attack is marked by strong cerebral symptoms, being ushered in by sudden and deep coma and profound unconciousness, or raving delirium. This was notably the case in the epidemic of 1853 in the locality where I was practicing. In those cases I bled from the arm or opened the temporal arteries and bled freely, without regard to quantity, till the brain was relieved. And I declare here, though I bled many, I did not lose a single patient thus treated. You may call this bold treatment, yea, hazardous, if you please; and certainly it would be so pronounced by *Reynolds* and *Flint*, and men of that school of philosophy ; but the result disproves the truth of their theory and teachings. Besides, there is no other prompt and efficient means of relieving this dangerously excited or fatally congested vital organ, and if the practice is bold, I reply that a physician's calling requires nerve and boldness to meet emergencies, and judgment to adapt his measures to counteract pressing dangers. To trust to revulsives and cerebral and cardiac sedatives in such cases were certain death. Besides, all this talk of late years against blood-letting, of drawing off the vital fluid in sthenic and inflammatory diseases, and the dangerous debilities and slow convalescences following the abstraction of blood, is sheer nonsense, having no foundation in fact, in sound reason, or correct physiology. It is a bug-bear, that serves only to make cowards of the timorous ; to put into jeopardy human life, and should be spurned by all intelligent and scientific physicians. I would like to speak more fully upon the subject of blood-letting, so important do I regard it in some cases, but I must refrain, lest I should be tedious. Having now gotten our patient's fears dispelled, his mind toned up, his brain relieved, his stomach and bowels cleared, and his skin moderately perspiring, we can further investigate his symptoms. We find that his tongue is furred, his saliva thick and clammy, his epigastrium and right hypocondrium tender with a sense of tightness, his urine scanty, his eyes injected, his temperature elevated, his pulse quick and respiration hurried, and pain in head, back, limbs. He has fever, and his symptoms point to *sepsis*, with a recognized tendency to rapid destruction of the mucuous coat of the stomach, structural change of liver, disorganization of the kidneys and decomposition of the blood. These are the prominent pathological changes going on in the system of a yellow fever subject, clearly revealed by the symptoms and corroborated by *post-mortem* examinations.

To meet these conditions, for an adult I usually prescribe 20 grs. calomel with 20 to 30 of quinine, divided into four doses, giving one every four hours, till all are taken. And this I do in the *hot stage*, as early in the attack as possible, provided there are no brain complications to oppose the use of quinine. Under the use of these, the fever and temperature subside, the patient becomes calm and comfortable, the urine more abundant ; a state of apyrexia has ensued. The bowels generally act spontaneously, passing off dark, thick, tarry stools. If they should not, use an enema of warm water with camphorated oil, or the infusion of senna and epsom salts will bring that result about.

This is generally all the medicine that I resort to. Within the first twenty-four hours I have pushed my active treatment and cured my patient, or else relieved his embarrassed and oppressed vital organs, and placed him in a condition favorable to a progressive return to health. By the calomel, I have aroused the liver to its normal functions and disgorged its vascular congestion, and soothed the irritated mucous coat of the stomach. By the quinine, I have quieted the turmoil of the organic system of nerves and given them strength, and thus have restored the several organs to their normal and harmonious work, and thereby counteracted the *septic* changes of the blood. The further treatment consists simply in good nursing—keeping the patient quiet in bed and comfortable, free from all excitement of body or mind ; the regular and careful administration of suitable nutrition ; the moderate allowance of good brandy, cool drinks, and no exercise or company under ten to twelve days.

Unfortunately, should the case not be seen sufficiently ready to admit of this decisive curative treatment ; or if seen, and the proper remedies were not used ; or if, from imprudence or unfavorable surroundings or constitutional proclivities, complications should develop, they must be met by rational modes of treatment, and not by reckless experimentation. Should nausea or irritability of the stomach manifest itself, a *fly blister* should be promptly applied over the epigastrium, and ice moderately allowed or cool water ; and, where no brain symptoms interdict it, small doses of morphine with mint water and bicarb. soda will answer a good purpose. And I have frequently used, with great advantage, small doses of creosote combined with a little morphine and bicarb. soda, in form of an emulsion.

If the patient is restless and sleepless from nervous irritability, morphine or dover's powder will be called for, to induce calm and refreshing sleep. If there be active cerebral congestion, or hyperæmia of the brain, in the second stage of the disease, cold or iced applications must be kept to the head, and bromide of potass. given internally. If a hemmorhagic tendency displays itself, or black vomit threatens, the free and liberal use of the muriated tincture of iron, conjoined with ice and good cognac, I have found to be of great advantage, rescuing patients almost from the jaws of death.

If suppression of urine should come on, which I regard as one of the most discouraging and fatal complications, I know of nothing better than dry cups over the kidneys, and frequent frictions with warm spirits of turpentine, mixed with tinct. digitalis, and the internal use of nitrate potass. in warm flaxseed tea.

Such, gentlemen, has been my general plan of treatment. I speak of it confidently, because it has given me the most gratifying results. These good results, which I have claimed as attainable by a judicious and timely treatment of this disease, may be regarded by some of you as extravagant and chimerical, being so opposed to almost universal experience. It was my purpose not specially to advert to my own practice and statistics, to verify my opinions on this point, for fear of being misconstrued; but, since writing the foregoing, I bethought that, at my advanced age and the necessarily few short years that at best I can remain in active practice, surely you would do me the justice to believe, that if I should be mistaken, my single and only purpose could be to advance the success of practical medicine and benefit suffering humanity.

I then state that, beginning with the epidemic of 1853, and with every subsequent epidemic, my experience has been uniformly the same and tallies with the statistics of 1878. In this latter year we made daily reports of our cases occurring, with certificates of death, to our health officer, setting forth location of case, name, age, sex, single or married, race, color, etc. That year our health officer was our respected ex-president, Dr. J. W. Dupree, who being present, can easily correct any misstatement, if I make any. My reports and certificates to him show that, in the space of two months, I treated between 600 and 700 cases, and that my deaths were 17 out of the whole number. You may call this good luck, or whatever else you please; still the fact remains, that I lost in 1878, out of nearly 700 cases of yellow fever, only 17 patients. And I verily believe, that whatever measure of success attended my professional efforts, would follow the practice of other medical men exercising the same careful thought and timely efforts that I gave to each of my cases.

Gentlemen, I have intentionally avoided quoting the opinions of different authors upon this subject, and contrasting their conflicting and opposite plans of treatment, because I did not wish to parade before this society what has been written by others, and already as familiar to you as to myself. My aim has been to give you my own thoughts in my own language and my own experience. If I have made my views clear and intelligible, I am satisfied. You can test them in practice, if you like. If you do it faithfully, correctly and *in time, which is all important*, you will soon disarm this scourge of its terror, reopen the obstructed channels of commerce and travel, and make our Queen City the delight of the whole land.—*N. O. Med. & Surg. Jour.*

### DENTAL THERAPEUSIS.

### CHAPTER XX IN PROF. JAS. E. GARRETSON'S SYSTEM OF ORAL SURGERY.

A carious tooth is to be saved through the character rather than by the quality of a plug. The expression of gold is one of compatibility with dentine; that only. It is a substance wholly without therapeutic meaning, save as such meaning lies in an ability to protect an exposed weak surface against external agents of offence. Teeth made up of solid, resisting stroma, will be well treated if invariably filled with gold. Preference assuredly is to be given this metal in instance of every individual case, cæteris paribus, where a plug is to show. It is also to be given where elegance and purity of expression come at all into consideration. In a word, it is desired to have understood that the teachings of this volume favor the employment of gold as a tooth-filling material whenever and wherever not contra-indicated. It is as well desired to have plainly expressed the view that fully one-half the operative dentistry of the day differs in no respect from a jeweler's work.

To be able to fill a tooth solidly and beautifully with gold, especially as contouring is concerned, is to have achieved a very creditable accomplishment; is not, however, to have learned anything scientific. What is done is not half so difficult as things being accomplished every day by workmen who think nothing at all of what they do.

Operative dentistry is not special surgery; it is art, not science. In this respect oral surgery is of little relation with dentistry; that art being viewed as a profession, whole in itself.

Whoever would treat and fill a carious tooth in relation with the laws of surgery is to treat and fill it in relation with indications. A filling of gold is an inert filling, it does nothing but stop a hole. Removal of a thoroughly well-made plug of gold, which has remained in relation with a cavity intact for many years, shows no change in the parietes of the cavity; the part remains as when brought in relation with the metal.

Other materials brought in contact with the parietes of a cavity in a vital tooth are found on removal to show changes; these have stopped holes, they have as well worked therapeutically.

A first consideration, as reference is had to selection of a tooth-filling material, may be instanced as referring to thermal conductivity. With some, with a great many teeth, such conductivity means nothing. with others, a great many others, it means inflammation of a pulp.

Gold is the most marked among the tooth-filling materials as a conductor. Where irritation is contra-indicated the metal is not judiciously to be used except in combinations. Where a cavity is large or a pulp nearly exposed, non-conducting substances are to underlie plugs made of gold; otherwise plugs are to be made of other materials.

Teeth are found where gold is unsuited because of its density; a soft tooth filled with cohesive gold, the mallet having been used, is oftentimes felt by the patient as possessed of a stuffed feeling; the discomfort being so great as to compel removal of the mass.

The opposite of gold, conductivity being considered, is gutta percha. Judgment plays the one against the other.

Soft teeth are most surely to be saved by a prophylaxis that considers a re-excitation of the calcification lying in the dental pulp. Agents used with this intent, named in the order of the excitant quality possessed, are chloride of zinc, copper, tin.

To recalcify a tooth means to harden it. To harden the parietes of a cavity of decay means to render the tooth resistive of external influences. Calcification arises out of judicious stimulation of a pulp; over-stimulation changes excitation to degeneration; defeats and perverts, consequently, the result of an intention.

To judiciously use oxychloride as a filling material is to possess measurement of the wants of the tooth to be filled. A majority of teeth can be recalcified. What, however, proves the equitable stimulation of one is over-stimulation to another. It is not to be denied that the chloride of zinc has destroyed more pulps than it has calcified teeth. No rule resides in the measurement of its use; proper employment of the agent lies in deductions arising out of experience.

In over-excitable teeth gutta percha takes the place of the zinc chloride; or, in instances, the floor of a cavity may be covered with it, or with a layer of oxide of zinc, and the oxychloride placed upon this. Where gutta percha is used the red variety is to be preferred.

Where gutta percha is selected as a material for foundation it is not unfrequently to have incorporated with it most advantageously fine filings of copper; or dust of the metal may be spread over the floor of the cavity and the gum used to hold it in place. Tin filings apply admirably in the same direction.

Oxide of tin, found in connection with all tin plugs, is a reliable medicament as the calcific process is concerned. Teeth from which the finest made gold plugs fall away by reason of secondary decay are restored to integrity through the use of tin. As a rule, all children's first teeth, and all soft teeth, are filled safely where tin is the agent employed. In many cases the removal of gold plugs and the refilling of the teeth with tin foil results in the preservation of a denture. After recalcification the tin is to be replaced with gold.

The progress of secondary calcification is to be measured by the occasional removal of a therapeutical plug. If it be found that the process is not advancing with sufficient sureness or rapidity, opportunity is afforded to remedy the default in the application.

Electrical disturbance is to have consideration. Viewing the matter apart from unsettled theories, it is undeniably the fact that a plug made of gold or amalgam, more particularly of the former, quickly becomes imperfect at a point where metal, gum, and tooth associate. Where, in filling such teeth, dryness was secured, the explanation of the deterioration is to be assumed as lying apart from original defect in manipulation. Such teeth are saved by making a neck plug of gutta percha; after this tin best applies.

Amalgam made with cadmium as a component when used in a tooth induces a condition of the pulp analgous to albuminoid degeneration. On the contrary admixture being made with copper the result is so peculiarly tonic that subsequent years will not unlikely show the pulp contracted to a thread, this arising out of the physiological effort made at calcification.

A concluding reference is to be made to the use of agents acting as parasiticides. No tooth is prepared for any, save an oxychloride, plug until a fungus-destroying application has been made to the cavity to be filled. Teeth of loose structure most particularly are infected with parasites. These are to be eradicated, for these it is which conduce much to leakiness, which leakiness, in its turn, has related with it the meaning of tooth or plug decomposition. Such decomposition being resultant of a battery made by the juxtaposition of two solids and a fluid.

As a parasiticide creosote most conspicuously recommends itself. It is to be used with every plugging material save those containing zinc chloride, this last being an agent of the same import. A cavity thoroughly saturated with creosote is rendered clear of all fungi.

### OBSTETRICAL EXPERIENCES.

Dr. David McWilliams, of Liverpool, in an abstract of 2,500 confinements "chiefly among the comfortable middle classes," states that he considers the forceps a great boon, always to be used with comfort and safety, without injury to the mother, and in *only one case* did he find craniotomy *necessary.* For over twenty years he has introduced the forceps into the uterus, often saving the child by that means, when the os was very narrow, but dilatable. He had only employed chloroform in the first stage to overcome rigidity ; in the second stage he often administered it till complete unconsciousness was produced, believing that the perineum may thus be frequently saved from rupture, an accident which will sometimes occur after every precaution. He has cured a complete rent, involving the sphincter, without operation, by rest, local cleanliness, and in the induction of temporary constipation by opium. He trusts in ergot especially as a preventative of flooding in cases where the pains are weak and the intervals long. He denies, on the evidence of distinguished travelers contrasted with the records of contemporary British practitioners, that puerperal mortality is the result of civilization. The truth is quite the other way, and by acting on increased knowledge, more lives will yet be saved.—*British Medical Journal.*

### HOANG-NAN ONCE MORE.

SIR: I have within a few days received a letter from the Rev, Father Etienne Brosse, from which I here extract a few paragraphs:

"I have read with pleasure the analysis you have given, in the *Medical Record* of March 12th, of my letter of January 27th. The abstract published is correct. Permit me, however, to further develop one point that was but briefly referred to, but which nevertheless appears to me to be of great importance. I allude to the action of hoàng-nàn as an intellectual stimulant. It sharpens the wits and renders the body more active and disposed to work. (*Il rend les ideés singulièrement faciles, nettes et vives*; *l'esprit est plus ouvert et gai et le corps plus alerte et dispos.*) This action is maintained all day after a single morning dose. Further, this stimulant action is not followed by an enfeebling reaction. The dose may be repeated daily for years without impairment of the general health. I have used the drug for the purpose indicated for four or five years, and several of my colleagues have had recourse to it in order to facilitate their labors both of pen and tongue.

"COCORITE, April 26, 1881."

Further interesting information concerning the drug will be found in a recent thesis by Baralt, entitled "Du Hoàng-Nàn et de son emploi contre la Lèpre," Paris, 1880.

Planchon has carefully examined the crude bark, but fails to find any characters that will enable him to distinguish it from the bark formerly known as the "false angostura." It will be remembered that at the beginning of the present century angostura (*cortex euspariæ*) was in high repute as a febrifuge, tonic, etc. In 1804, however, a quantity of bark appeared in the European market that purported to be angostura, but the use of which was followed by serious and even fatal results. Subsequent investigation revealed the fact that this spurious angostura was nothing more than the bark of the *strychnos nux vomica*, the effects of which appear to be similar to, though probably not identical with those of the officinal portion of the plant. It may turn out ultimately that the hoàng-nàn of to day is the same bark reintroduced into commerce under its Indian name.

for nux vomica bark under its own name is not, I believe, in market at present. However this may be, there can [be no doubt as to the activity of the substance in question, and little doubt as to its usefulness in several directions, although it is questionable if future experience will give it the high place that its missionary friends appear to accord it.

In this connection it may be proper to state the young leper exhibited by me at the Academy of Medicine in January last has improved while taking hoàng-nàn, in the most striking manner, both as to appearance and in general condition, and is now so far recovered that he works daily at the oar as one of the crew of the barge that plies between Charity Hospital and the city.

June 9, 1881.

Respectfully yours,

HENRY G. PIFFARD, M. D.

*Medical Record.*

### LEPROSY IN KEY WEST, FLA.

A correspondent of the *Tribune* recently sent what appears to be a somewhat sensational account of leprosy at Key West, Fla. He says: Some three years ago, while on the island of Key West, Fla., I was surprised to find leprosy there in all its horror, and making bold to speak of the matter, I was immediately condemned for attempting to injure the commercial prosperity of the place, and particularly in destroying its reputation as a resort of Northern invalids. The reputation seemed to dwell only in the minds of the sanguine, for but few invalids would risk their lives in a place like Key West, notorious for its yellow fever. However, I was stationed at Key West for six or seven years, and can confidently state that there are many cases of leprosy on that island, whole families being afflicted with it, and in several instances it was found in its worst form. No provision is made for the unfortunates; they dwell surrounded by the masses, intermarrying, and die. To strangers who happen to discover the truth, it is a source of continual anxiety, for one does not know when wash-clothes may be returned impregnated with the poison. This disease has been thoroughly and undeniably traced to the English possessions in the adjacent islands. I can assure you if a cautious and honest investigation is made in that locality the result would astonish the people of this country. A selfish spirit prompts the leading people of that beautiful but poisonous island to disguise the real state of affairs existing there, and I would take but little comfort in smoking a genuine Key West cigar, knowing of the existence of these horrible facts. The summer of 1880 in this island is locally notorious for its yellow fever horrors; still the press of the country would lead one to suppose there existed there but a few passing cases. Only government officials, like myself, know of these horrors; and strangers have been repeatedly lured there only to find a grave. I have no personal interest in condemning Key West, but am only possessed of a desire to warn the ignorant and confiding, and condemn the wicked and selfish, who, being saturated with the poison themselves, are reckless for gain and indifferent of results.

### LEPROSY IN LOUISIANA.

In connection with the effort initiated by the New York Academy of Medicine, to investigate the subject of leprosy in the United States, the report of the Louisiana Board of Health for 1880 contains some interesting statements. It gives a history of the progress of leprosy in that State during the last and present centuries. The disease was brought in 1680 to the West Indies by the negro slaves, and thence to Louisiana. In 1778 this disease was so prevalent among the blacks, together with the African elephantiasis, and another equally horrible, named yaws, peculiar to the Guinea negroes, that a hospital for lepers was established in New Orleans. At the present time the majority of the lepers in that city are found to be whites, of French, German, and Russian extraction. It has shown itself to be hereditary, and probably contagious.

An Italian Catholic priest, who attended cases of leprosy in the Charity Hospital, is now dying of it in the same house. Yet New Orleans, it appears, has no separate asylum for these incurable patients, and they are received into the Charity Hospital and placed in the crowded wards to scatter death.

The president of the Board of Health has made a personal investigation into the extent of this disease, even venturing into the swamps of the lower Bayou Lafourche. This whole district, he states, is several feet lower than the turbid bayou, sloping back into cypress swamps liable to constant overflow from crevasses. The poor Creole inhabitants live in low huts surrounded by wet rice fields, living upon fish and fish-eating birds. They are separated from the rest of the world and have intermarried for generations. So impregnated with disease is this remote region that some of the exploring party were struck down on reaching it with violent hemorrhages and fever. Of all foul corners of the world it is the fittest for the disease most dreaded by man since the beginning of the world to hide with its prey. Below Harang's Canal, President Jones found Asiatic leprosy existing in different generations of six families. Some of these wretched creatures have been driven out from human habitation, are living apart in the swamps, dying of decay. It was impossible to make a correct estimate of the cases, as a rumor spread among them that the searching party had come to carry them off to an uninhabited island of the sea, and they hid themselves. their friends, too, refusing to tell their names or number.—*Medical Record.*

### LISTERINE, THE NEW ANTISEPTIC PREPARATION.

We are glad to call the attention of our readers to a new and valuable contribution to antiseptic surgery. It is called listerine, and the thought suggesting the name is indeed a happy one. It is a combination of the essential constituents of thyme, eucalyptus, baptisia, gaultheria, and mentha arvensis. Besides these, each fluid dram contains two grains of refined and purified benzo-boracic acid. These substances, carefully prepared and combined in a solution of uniform strength, cannot fail to do good service in the treatment of all affections requiring an antiseptic.

The preparation is convenient, safe and agreeable. Locally it will be found of real value as a dressing for wounds, ulcers, and abscesses. It may also be employed as a constituent of solutions for atomization in lung affections and of gargles in throat diseases, while internally it must prove efficacious in all forms of fermentative indigestion.

Surgeons and physicians who have made use of any of the well-known ingredients of Listerine can attest their value, and will not fail to appreciate the advantage of having them always at hand in a suitable combination.—*Louisville Med. News.*

### PREPARATION AND USES OF BENZOATE OF CALCIUM.

Mr. Jas. T. Shinn gives directions for the preparation of this salt in the *American Journal of Pharmacy*, April, 1881 :

The proportions of benzoic acid and calcium in the benzoate are 113 to 20, or of the crystallized acid and carbonate of calcium, 122 to 50. Hence the following formula for its manufacture :—

| | | |
|---|---|---|
| ℞. | Benzoic acid, | ℥ iv gr. xxxij. |
| | Calcium carb., | ℥ j ʒ v ℈ j. |
| | Boiling water, | Oiv or q. s. |

Mix the acid and precipitated chalk thoroughly, in a large mortar, and add water gradually to allow most of the carbonic acid gas to escape and prevent frothing over of the liquid. When the combination has taken place, or nearly so, the mass is transferred to a porcelain dish and dissolved in the remainder of the boiling water, with the exception of a slight excess of carbonate of calcium. Filter, while hot, into a shallow dish, when crystals will form on cooling. The mother liquors may be evaporated twice more and yield more crystals, the whole product being about ℥ iv, ʒ v.

The salt is in feathery crystals, of a silky lustre, odorless, with but slight, rather alkaline, taste, and is soluble in about 24 parts of water. It may be dispensed either in capsules or solution, a very good form of the latter being :—

| | | | |
|---|---|---|---|
| ℞. | Calcii benzoat., - - - - | gr. cxxxviij | |
| | Aquæ destillat., - - - - | fl. ℥ vj | |
| | Syr. aurantii, - - - - | fl. ℥ ij. | M. |
| Ft. mist. | | | |

This makes a solution, by the aid of heat, containing eight grains to half a fluidounce, which is the usual dose.

At a meeting of the Philadelphia County Medical Society, Drs. A. H. Smith and O'Hara spoke of the great benefit this preparation had been in cases of albuminuria during pregnancy, and, as the salt may be called for and is not on the price lists of the chemist, the above formula for its preparation may be useful. The first crop of crystals can be taken out and dried on bibulous paper within an hour of the receipt of the prescription by the apothecary.—*Med and Surg. Reporter.*

### DOES EXCISION OF THE TONSILS IN A MALE INFANT DESTROY VIRILITY?

Dr. Penrose (*New York Med. Gazette*) says: I would never excise a male child's tonsils without explaining the possible effects to the parents, for I saw some years ago a statement made by a writer in one of the foreign quarterlies that amputation of the tonsils for the cure of chronic tonsilitis was sure to destroy virility in a man. Some time after reading the article in question a medical friend happened to drop in, and, not at all satisfied in my own mind regarding the accuracy of the writer's statements, I put the question directly to him: "Do you believe that amputation of the tonsils destroys a man's virility?" "O, no, Doctor," said he, "that is all trash; why I had my tonsils cut when I was a child." Gentlemen, my medical friend had been married twenty years. Though his wife is an unusually healthy-looking woman, she has never had a baby.

### THE USE OF THE LANCET.

I have a bit of a confession to make, and also a request. I confess being so much of an old fogy, that for twenty years I have carried the *deadly thumb lance* in my pocket, and what hurts me most, have not used it as often as I ought. During the four years of my pupilage, I saw my preceptor use it once only. For two years after graduating I did not use it at all. Since then I have used it more and more each year, and can truthfully say, that I have never seen a single bad effect from its use; but have often had to lament in sack-cloth and ashes that the prejudice of patients, or the opposition of a consultant, has compelled me to witness many a death which might have been avoided, and many a long convalescence which might have been shortened, by an early and free use of the lancet. My fogyism extends even to the use of calomel in many acute inflammatory diseases. The request mentioned is, that some vigorous writer (or rather thinker) would do for calomel what Dr. Corson is doing for the lancet. It is a belief that fire will never melt out of me, that a proper use of these two agencies would completely cure many men and more women of acute inflammations, who, under the expectant and dish-water treatment, only so far recover as to be thereafter daily reminded that it has left them a legacy of which they cannot dispose. In the *Reporter* of April 30th, Dr. Stinson asks, "When bleeding is indicated, what practitioner will refuse to resort to the lancet?" Why, Dr., that is just the very point. Nineteen out of twenty fail to see the indication; and the chances are vastly in favor of the twentieth one being afraid to act in opposition to the *great minds* of the profession.—F. R. Millard, M. D., *in Med. and Surg. Reporter.*

### VACCINATE AND REVACCINATE.

Our English cotemporaries with one accord admit that "what looks very like a smallpox epidemic is now raging in London."

In one locality alone there are more than three hundred cases under treatment, while in others new pest-houses must be built to meet existing necessities. Close and ill-ventilated quarters in various parts of the city form hotbeds of infection, which keep the disease alive and in a condition to spread far and wide. "The authorities are apathetic and sanitary inspection is a farce."

In the face of all this danger the anti-vaccination societies are getting in their work. Vaccination being compulsory is submitted to with doubtful grace, but revaccination meets with stubborn resistance. By means of public meetings and the free circulation of anti-vaccination literature the prejudice of the ignorant is wrought up to a menacing pitch. There have already been outbreaks of violence, and mob-law threatens to disarm the sanitary authorities and turn the city over to pestilence and death.

With a view of counteracting the influence exerted by these enemies of the public good, and neutralizing the effect of their pernicious literature, the National Health Society has issued a pamphlet calling attention to the prophylactic power of vaccination, insisting upon revaccination, and giving a number of facts which cannot fail to carry conviction to all who will give them an unprejudiced hearing. Twenty thousand copies of this pamphlet have been circulated, and many more are ready to be sent out.

This publication makes it plain that vaccination is the only available means of protection against smallpox; that with due care in the performance of the operation no risk of injurious effects from it need be run ; that before its discovery the mortality from smallpox was forty times greater than it is now ; that since vaccination has become compulsory in England the death-rate from smallpox is one-half what it was in the previous sixteen years ; that in the London Smallpox Hospital the records show a rate of mortality of less than one per cent. for well-vaccinated persons against a rate of thirty-five per cent. for the unvaccinated ; and that the degree of protection is in direct proportion to the thoroughness of the vaccination.

Is it not strange that in the very land of its origin vaccination should find its most bitter enemies ; and that, coupled with their senseless opposition, there should exist such indifference on the part of those entrusted with the carrying out of this sanitary measure that a smallpox epidemic is not only made possible, but that its horrors are again likely to be visited upon London ? Truly, "the mills of the gods grind slowly."

On this side of the Atlantic vaccination is certainly held in better favor, and its beneficent claims are for the most part acceptable as a verity. But there begin to appear on our horizon the ominous fringes of that transatlantic cloud, whose black shadow may yet perforce sweep over the land. New York can boast an anti-vaccination society ; and though no respectable opposition to vaccination is to be found among the molders of our professional thought, we now and then see ridiculous diatribes denouncing the measure in the columns of eclectic medical journals and gratuitously-circulated pamphlets.

Our sanitary boards are not over-zealous in seeing to it that the unwashed shall not be allowed peaceably to breed and circulate smallpox ; and it is among the possibilities of the time, to say the least, that we may yet have a taste of that bitter potion which our brethern across the sea are now forced to swallow.

Before the discovery of vaccination smallpox was more "terrible than an army with banners." Cholera and plague found lines beyond which they could not pass. Not so with smallpox. No alitude, no isothermal line could mark the limit of its march. It overstepped all bounds, it scaled the loftiest heights ; it blew its pestilential breath into the most salubrious and otherwise secure retreats of men, and laid its loathsome blight upon the fairest forms. Jenner came forward with his great antidote, and under its protecting power the civilized world has for more than a century enjoyed comparative immunity from the ravages of this disease. Medical philosophers have believed that, properly applied, the remedy would annihilate the pest, and have ceased to speak of a smallpox epidemic except as a thing of the past.

It now begins to look as if this might be an illusion, and we are called upon to face the ugly fact that ignorance, indifference, and misguided judgment can in one community at least cripple the wise in the carrying out of protective measures, and so open the way for a general onslaught of the disease.

Perhaps a visitation of smallpox, such as punished the inhabitants of medieval times, when it depopulated cities and spread desolation over the nations, will be necessary to bring the people of these days to a full recognition of their duty in the matter of vaccination.

It is not enough to know, we must *do* our duty. Knowledge may keep the mind in healthy exercise, and lead us to build theories and devise schemes for the promotion of sanitary science, but nothing short of that wisdom gained by experience from under the scourging hand of the pestilence will impel us to put them into effective practice. New Orleans was content to abide in filth, and Memphis to dwell upon the banks of an open sewer, till yellow fever had terribly punished the one and wrought ruin to every interest of the other. —*Louisville Medical News.*

### SOME POINTS IN THE TREATMENT OF BRAIN DISEASES.

1. *Tumor of the right cerebral hemisphere.* A merchant, aged 32, married and father of two healthy children, without any signs of syphilis, and who furthermore denied ever having been infected, was seized, apparently without cause, with vertigo and vomiting. Headache, disturbed sleep and delirium at night supervened. The memory became impaired. Optic neuritis, with choked papilla and extravasations of blood ensued, loss of power in the left arm and leg followed, but no reeling gait. Sensation was normal, likewise reflex from the skin, the tendon reflex being slightly exaggerated. Tumor of the right hemisphere was diagnosed, and although absolutely no sign of syphilis existed, the antisyphilitic treatment (mercurials with iodide of potash) was instituted. The effect is described as magical—the vomiting disappearing after the first dose, and the progress continuing to complete recovery.

2. *Diabetes insipidus.* A gentleman, aged 37, who had lived for many years in the tropics, and had suffered with persistent diarrhae, suffered from cerebral exhaustion with polyuria, so that as much as 90 ounces of urine on an average was passed daily. It was of low specific gravity, and contained nothing abnormal. Acting upon the supposition that the trouble was due to lesion of the medulla oblongata, the constant current was applied to the back of the head. After one application the polyuria entirely disappeared.

3. *Melancholia.* A woman, aged 28, became afflicted with melancholia after her confinement, which resisted all therapeutic measures, until the constant current was applied over the occipital lobes with voltaic alternations. After 20 applications she began to mend.

4. *Auditory delusions.* An epileptic patient was suddenly seized with auditory delusions, hearing voices calling him, speaking about his pecuniary affairs and his health. The constant current was applied over the superior temporo-sphenoidal convolutions for five minutes, whereupon the delusions immediately vanished, never to return.

5. *Corpus striatum hemorrhage.* A widow, aged 60, was attacked with symptoms of hemorrhage into the region of the right corpus striatum. As soon as the ankle clonus showed itself the constant current was applied to the head. This occurred on the 27th day. After 8 days, during which 4 applications were made, the paresis of the muscles of the trunk had entirely disappeared and the mobility of the leg improved. After 4 months the patient was cured, although slight paresis and rigidity in the arm and hand remained behind.

6. *Softening of the left hemisphere from embolism of the middle cerebral artery.* A lady, aged 40, was seized with a stroke, which robbed her of her speech and the use of her right arm. Ten years after that she came under treatment. Distinct aphasia was present, so that only very few words could be pronounced. Slight paresis of the lower facial muscles was present and paralytic rigidity of the right arm and leg. Ankle clonus was well marked. The general health was good, except that the menses had been absent for three years, since when she has had periodical headaches. The same treatment was pursued as in the former case, and continued for six months, whereupon marked amelioration ensued. The rigidity of the leg had entirely disappeared, so that the patient walked with ease. The arm had likewise been much bettered, but the most progress was observed in the speech. The menses returned after a few weeks' treatment, and the headaches entirely disappeared.—*Pacific Medical and Surgical Journal.*

### OPTHALMOLOGY AND OTOLOGY.

### NASO-CRANIAL OSTEITIS OF SYPHILITIC ORIGIN.

The following is a resumé of a lecture on the above subject by Professor Fornier :

1st. If, in the vast majority of cases, syphilitic nasal osteitides do not threaten life it violates in this respect the ordinary rule. This variety, osteitis of the roof of the nasal fossæ, *naso-cranial osteitis*, owes its special gravity only to its location.

2d. The danger of this naso-cranial osteitis is a repetition of the irradiation towards the organs contained in the cranial cavity, an irradiation showing itself anatomically by diverse lesions, the chief and most frequent being meningitis, encephalitis, abscesses of the brain.

3d. Clinically, these complications present themselves under two forms :

(*a*) A chronic form, characterized by vague symptoms of slowly progressive encephalitis with a brusque or apoplectiform termination.

(*b*) An acute form, characterized by symptoms of a marked, incomplete, irregular, but rapidly fatal encephalitis.

4th. These cerebral complications only rarely remain absolutely latent as a clinical expression and end unexpectedly in a sudden manner, by fulminate apoplexy.—*Annales des Maladies de l'Oreille.*

